# Elements of Physiology

**Published:** 1852-03

**Authors:** 


*Elements of Physiology, including Physiological Anatomy.* By WILLIAM B. CARPENTER, M. D., F. R. S., F. G. S. Second American, from the Second London Edition.

No one can compare the recent editions of Dr. Carpenter's works with those published eight or ten years ago, without being struck with the great advance which has been made in physiological science since that period. The omission of many statements which appeared in his former editions, and the modification or entire alteration of others, result necessarily from the peculiar character of his writings; — which are distinguished, as the author himself expresses it, not so much by the amount of original matter they contain, as by the degree in which "the knowledge of many has been orderly and methodically digested and arranged, so as to become attainable by one." Dr. Carpenter's works, indeed, contain but very few scientific facts which have been contributed by the author. Those portions which are original with him, consist almost entirely of the arrangement and comparison of previous observations, and the application or extension of scientific principles, already deduced by others. Dr. Carpenter, therefore, is not himself responsible for any statements of fact; and the alterations in these statements which are required by the progress of physiological science may rather be considerd as enhancing the value of his later editions, than as implying any essential deficiency in those which have preceded.

Of the three physiological treatises for which we are indebted to this author, the present is intended more particularly for those commencing a course of physiological study. Its object is to give the student a knowledge of "those principles of Physiology which are based on the broadest and most satisfactory foundation, and to point out the mode in which these principles are applied to the explanation of the phenomena presented by the living actions of the human body." It does not claim to give the student a full account of these phenomena, but rather "to prepare him for the more detailed study of the latter" which will be necessary in order to complete his medical education. The book is principally valuable, therefore, for the broad and comprehensive view of the science of Physiology which it presents, to the mind of the student, and for the assistance which it gives him in comprehending the connexion between its different departments. He is thus enabled to see the simplicity with which the vital operations are performed in the lower orders of organized beings, and to trace their gradual complication in the higher classes. He learns that the organic processes of nutrition, secretion, and reproduction are not essentially distinct from each other, but are to be considered only as different modifications of the same vital power;—that all these various forces influence each other, that they are often mutually convertible, and that there is, in fact, a "correlation" of the vital forces,—sensation, nutrition, contractility, &c.,—precisely analogous to that which had been previously shown to exist between the physical forces,—heat, motion, electricity, magnetism, &c. It is exceedingly desirable that the student should not remain destitute of this kind of knowledge. A mere mass of facts, thrown together in a promiscuous heap, cannot constitute true scientific information. Such science as that is merely "another kind of ignorance." It is only when we comprehend the relations between these facts, and are enabled to arrive at some general idea with regard to them, that we can be said to possess any knowledge worth having. Probably there is no living physiologist better qualified to point out these relations than Dr. Carpenter. Still we should never forget that, although the ultimate object of scientific research is to arrive at the deduction of general laws, this can be safely accomplished only by means of the collection and arrangement of solid facts;—and that it is essential to success that these facts should be absolutely correct and reliable. And there is one fault which is, perhaps, unavoidable in a work constructed on the plan of the "Elements of Physiology." The author, relying mostly on the accounts of others for his statements of fact, — which accounts are often more or less conflicting, — is unable to speak of them with that confidence which would result from a personal familiarity with the subjects. This gives rise to many vague and uncertain expressions in the course of his book, the constant recurrence of which produces a sense of dissatisfaction in the mind of the reader. One does not like to be told that "from recent observations it would seem" that so and so takes place; — that "it appears" that such "may be" the case; — or that "there seems adequate reason for the belief," &c. &c. Every reader, though he may be even a student in his first year, has a right to know, not only the facts of physiology, but also something of the methods by which those facts have been ascertained. Perhaps it would be more proper to say that no student should be *allowed* to receive a physiological statement, without at the same time, learning the grounds which the author has for making it. He will not then be at the mercy of every new theorist, and his progress will be much more satisfactory, though perhaps not quite so rapid. J. C. D.

